# Supplementary figures and images for: Associations between specialty care and improved outcomes among patients with diabetic foot ulcers
Source: PLoS One. 2023 Dec 19;18(12):e0294813. doi: 10.1371/journal.pone.0294813 (PMC10729988; doi:10.1371/journal.pone.0294813)

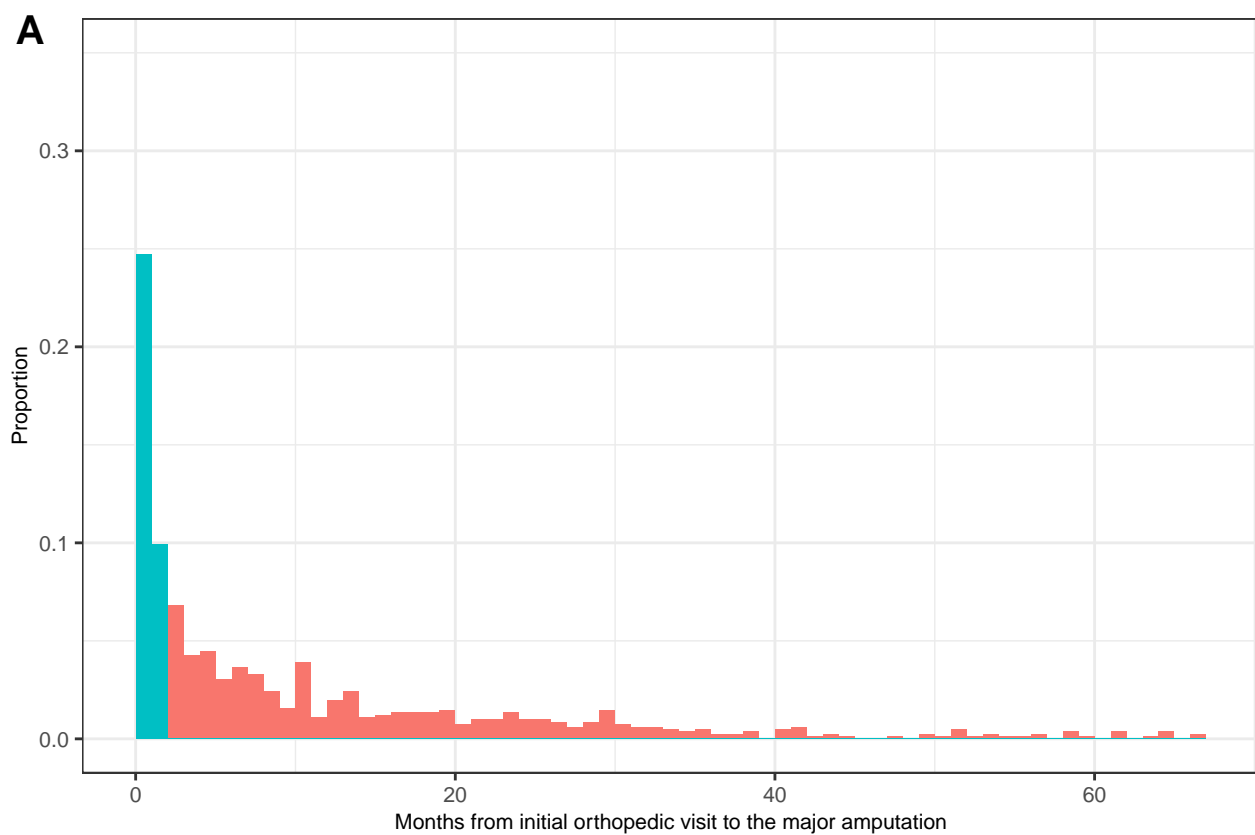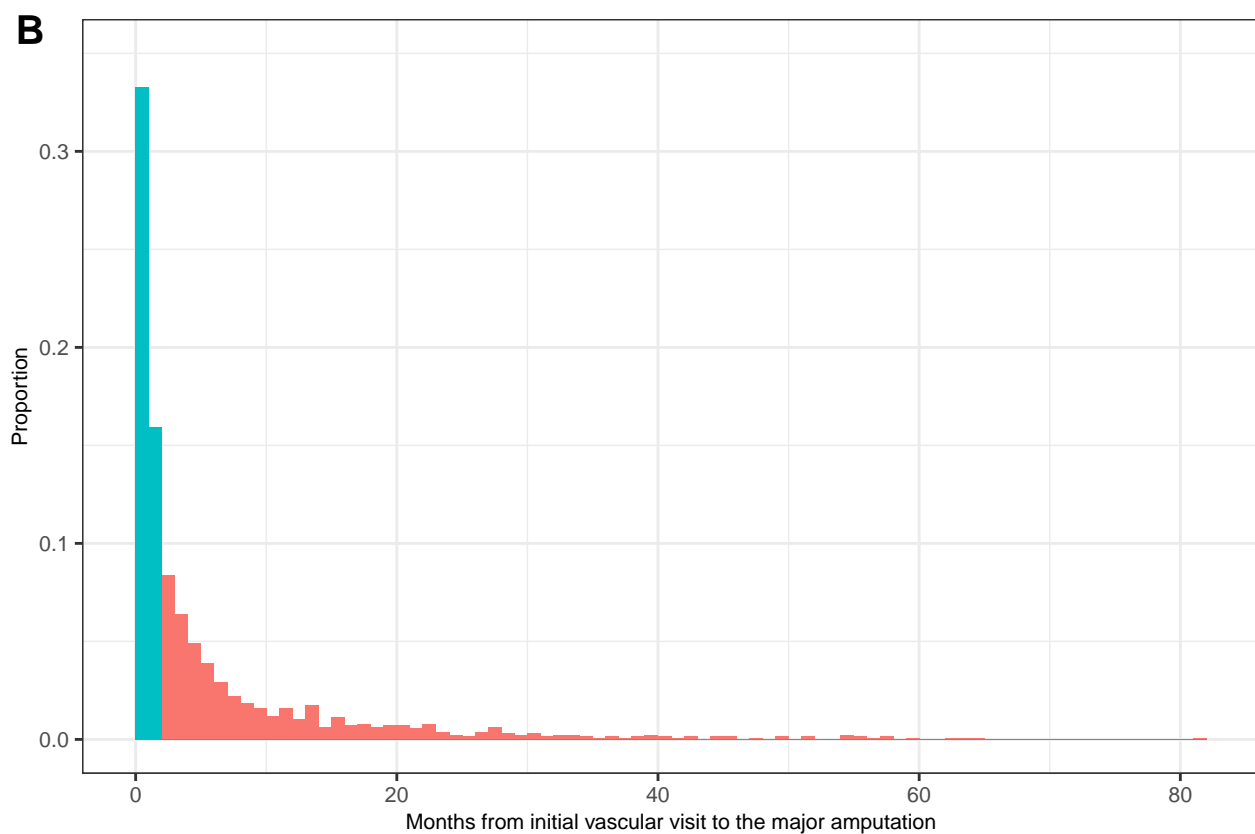

Supplement: S1 Fig — (PDF) [file pone.0294813.s001.pdf]

**A****Early stage ulcer stratum**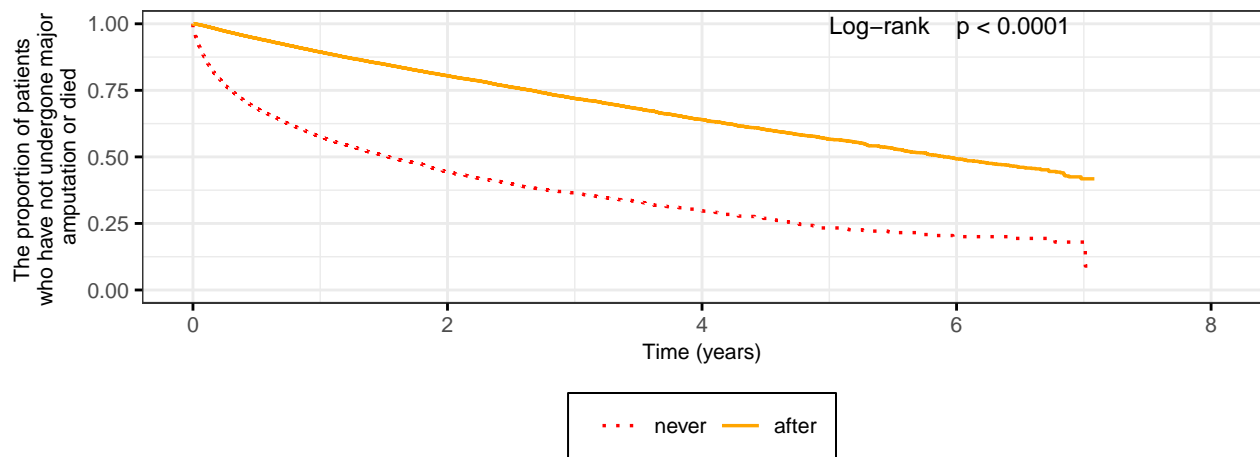**B****Osteomyelitis stratum**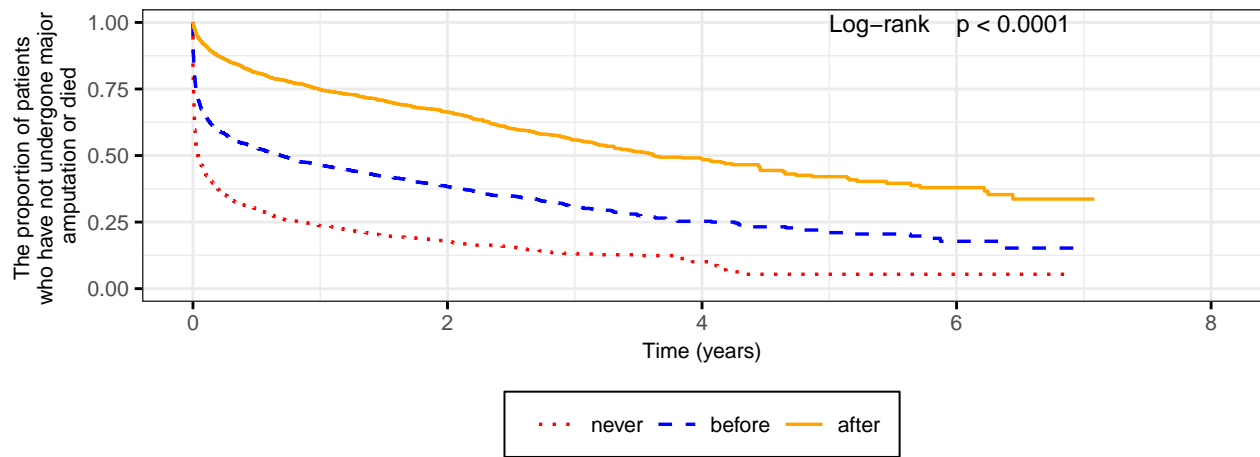**C****Gangrene stratum**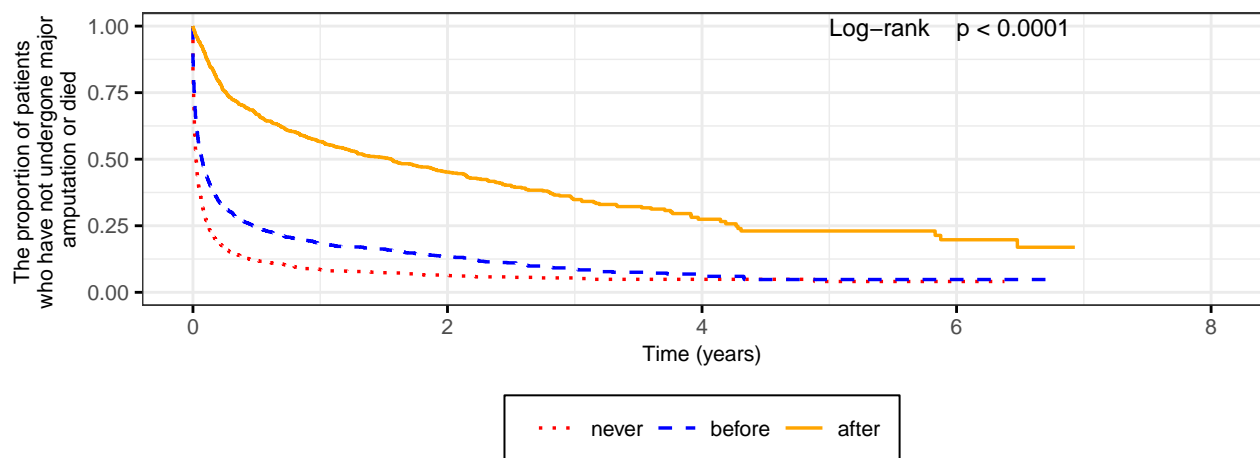

Supplement: S2 Fig — Within each stratum, patients were characterized as having never received specialty care, having received specialty care before the diagnosis of osteomyelitis or gangrene (which applied to some of the patients who entered the cohort with a less severe ulcer and progressed to osteomyelitis or gangrene), after entering the stratum (i.e., being diagnosed with an early stage ulcer, osteomyelitis or gangrene, respectively). (PDF) [file pone.0294813.s002.pdf]

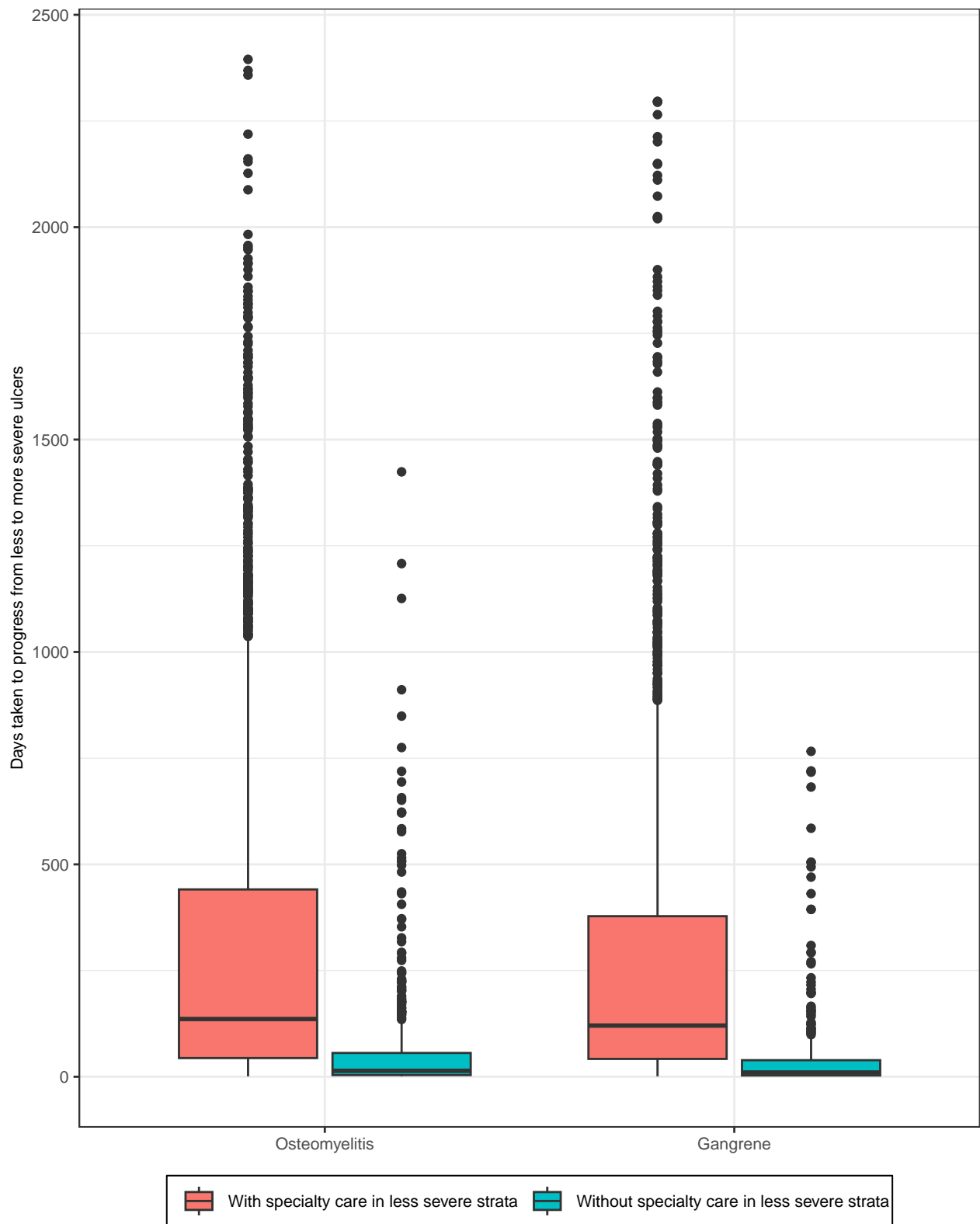

Supplement: S3 Fig — Analysis is based upon the most severe ulcer stage that a patient developed during the study period, either gangrene or osteomyelitis. (PDF) [file pone.0294813.s003.pdf]

**A** Osteomyelitis stratum

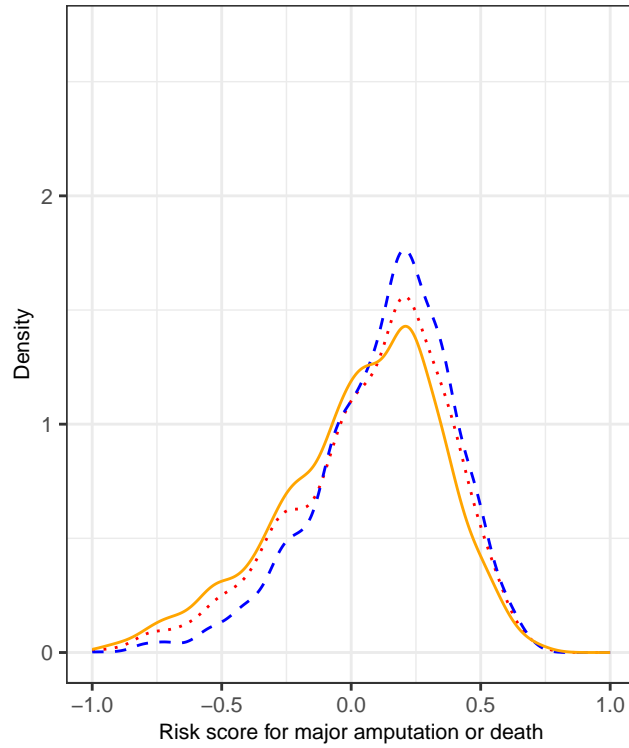

Osteomyelitis stratum

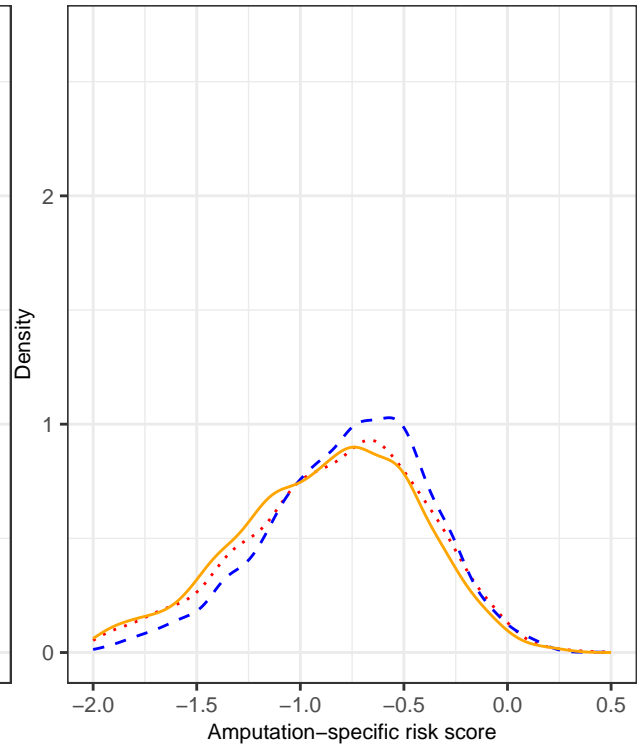

**B** Gangrene stratum

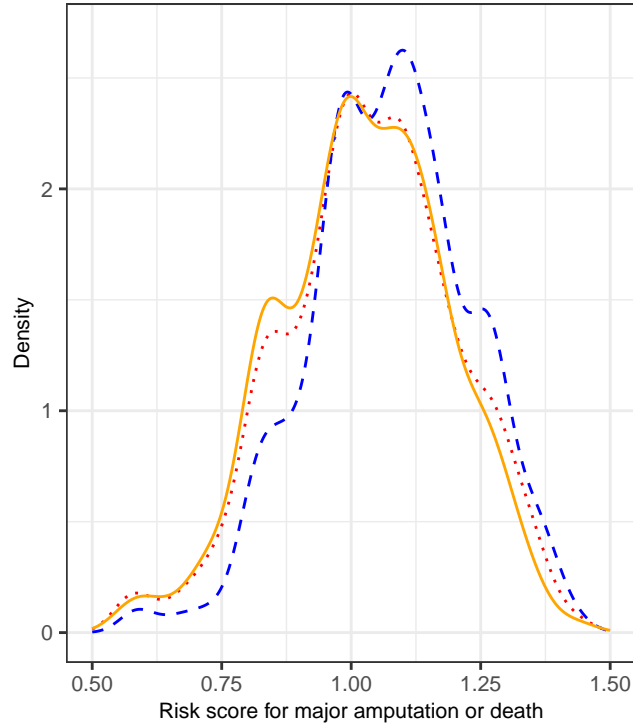

Gangrene stratum

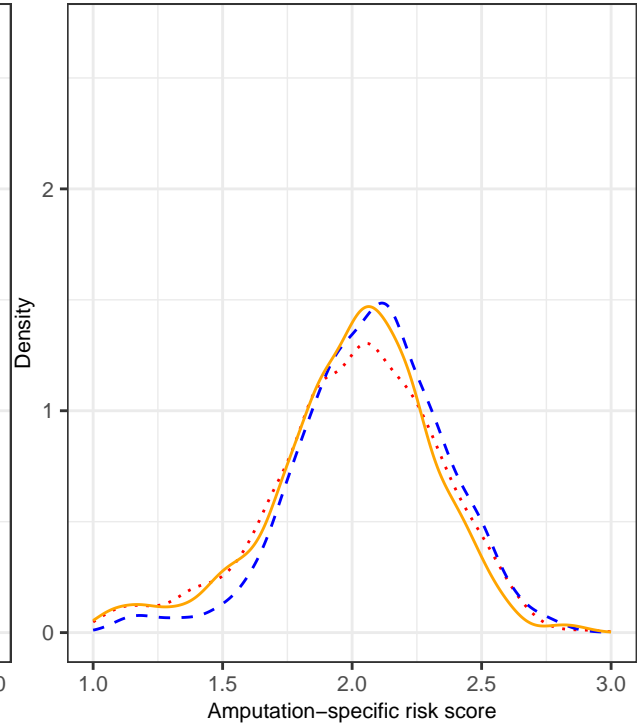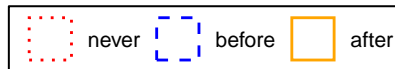

Supplement: S4 Fig — Red corresponds to those who never received specialty care. Blue corresponds to those who received specialty care prior to ulcer progression. Yellow corresponds to patients who received specialty care after diagnosis of either osteomyelitis or gangrene. (PDF) [file pone.0294813.s004.pdf]

**A****Early stage ulcer stratum**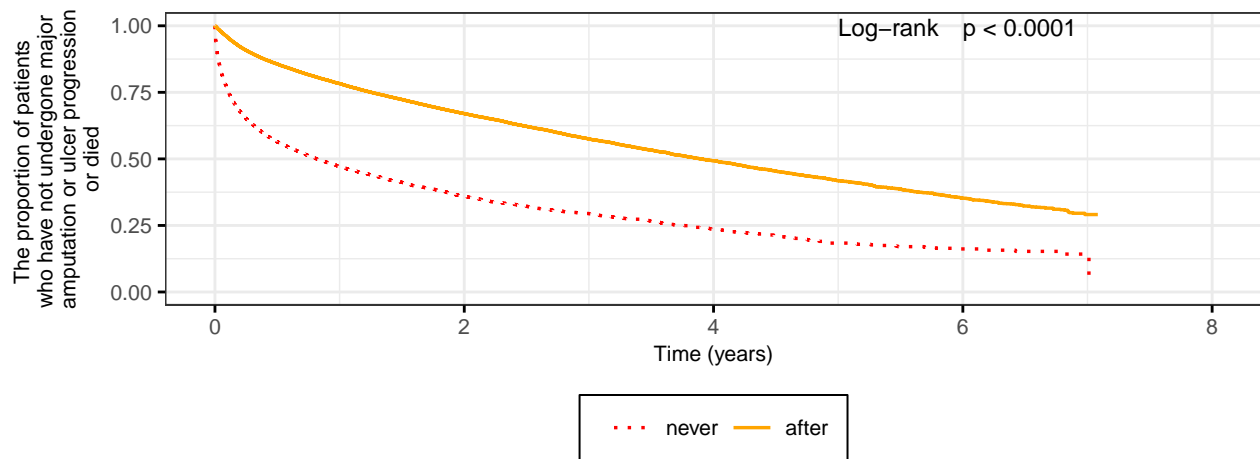**B****Osteomyelitis stratum**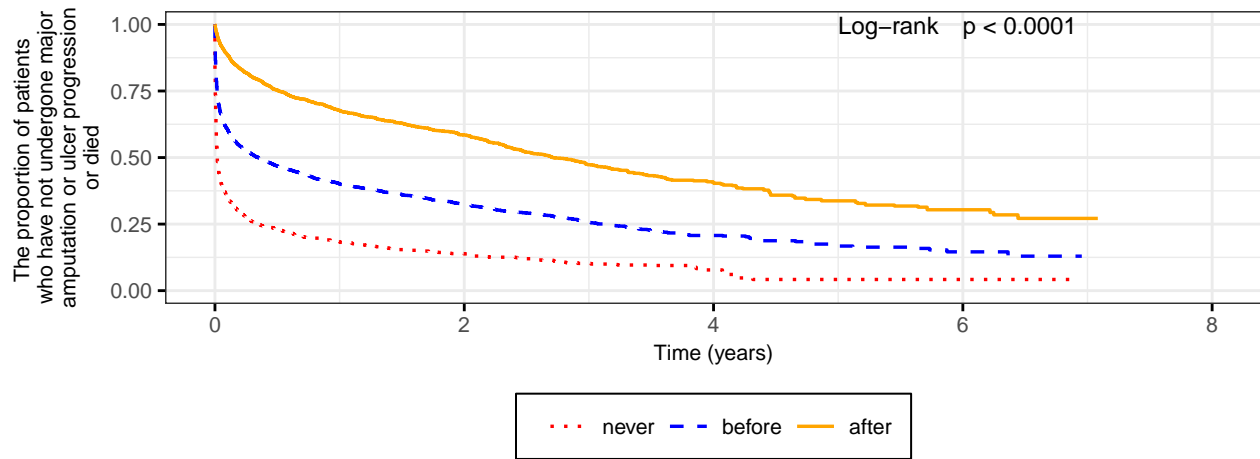**C****Gangrene stratum**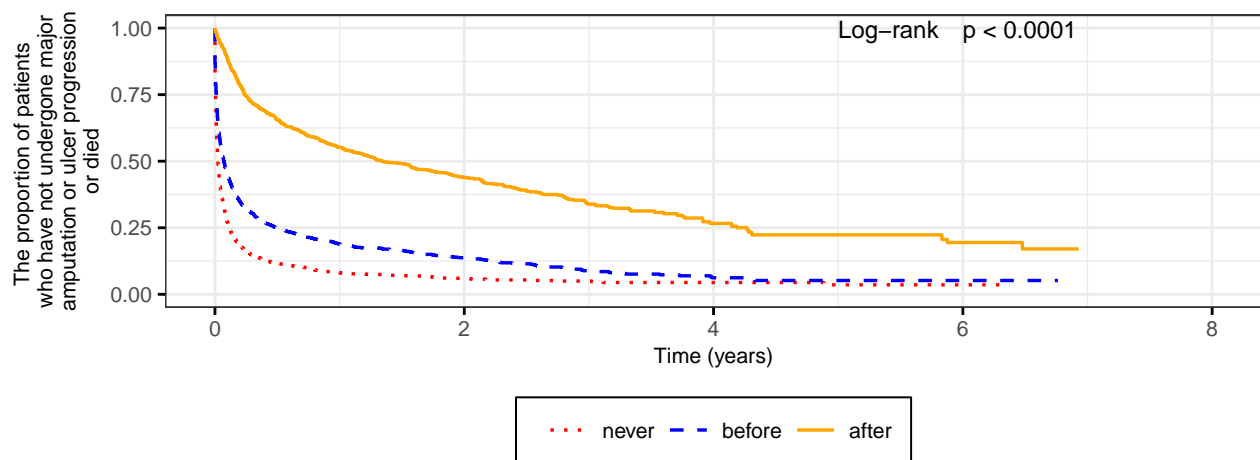

Supplement: S5 Fig — Within each stratum, patients were characterized as having never received specialty care (red), having received specialty care before the diagnosis of osteomyelitis or gangrene (which applied to some of the patients who entered the cohort with a less severe ulcer and progressed to osteomyelitis or gangrene; blue), or after entering the stratum (yellow). (PDF) [file pone.0294813.s005.pdf]

**A****Early stage ulcer stratum**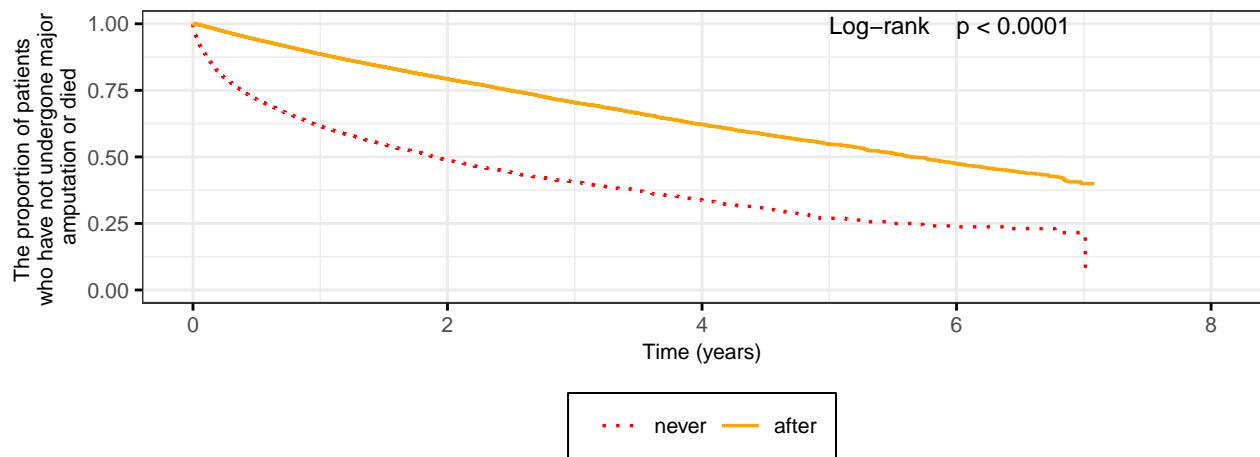**B****Osteomyelitis stratum**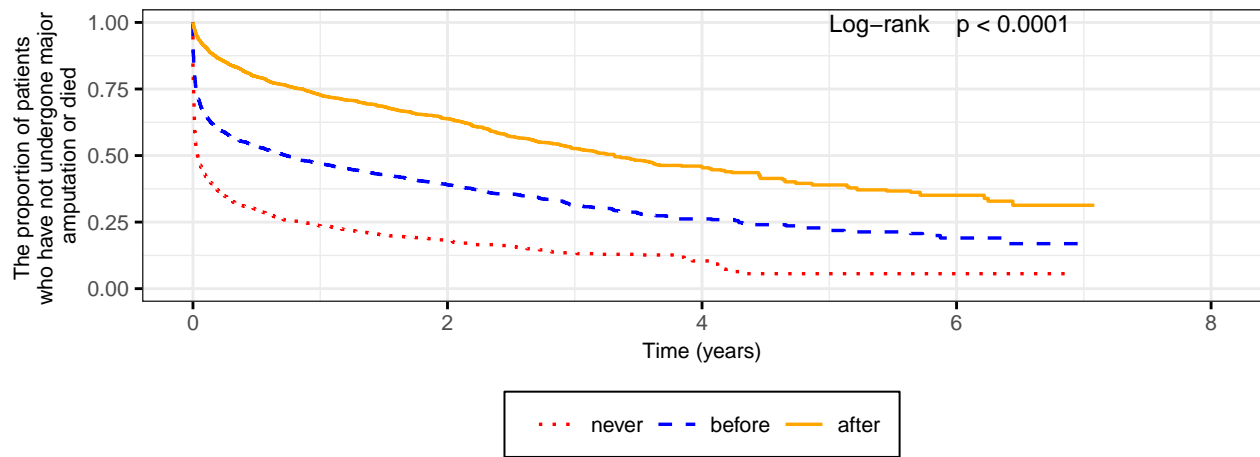**C****Gangrene stratum**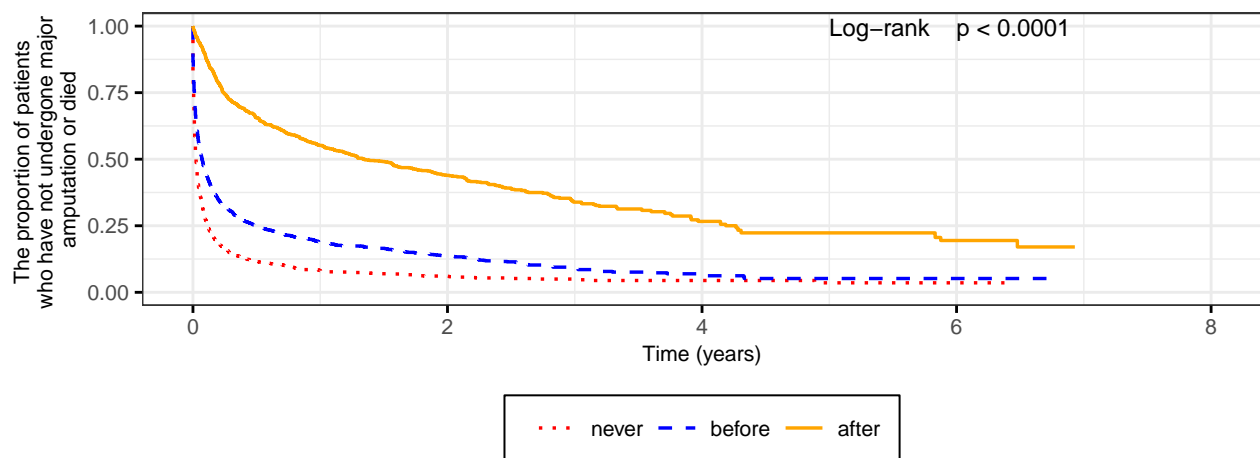

Supplement: S6 Fig — Within each stratum, patients were characterized as having never received specialty care (red), having received specialty care before the diagnosis of osteomyelitis or gangrene (which applied to some of the patients who entered the cohort with a less severe ulcer and progressed to osteomyelitis or gangrene; blue), or after entering the stratum (yellow). (PDF) [file pone.0294813.s006.pdf]
