# Supplementary material for: Associations between specialty care and improved outcomes among patients with diabetic foot ulcers
Source: PLoS One. 2023 Dec 19;18(12):e0294813. doi: 10.1371/journal.pone.0294813 (PMC10729988; doi:10.1371/journal.pone.0294813)
Supplement: S1 Table — (DOCX) [file pone.0294813.s007.docx]

| **S1 Table.** **International Statistical Classification of Disease and Related Health Problems, version 9, codes (ICD-9 codes) used to generate variables for the current study.** | |
| --- | --- |
| **Variable** | **ICD-9 Codes** |
| Diabetes | 250.xx |
| Foot ulcer severity |  |
| Early stage foot ulcer | 440.23, 707.1x, 707.9 |
| Osteomyelitis | 730.07, 730.17, 730.27, 730.97 |
| Gangrene | 0.40.0, 440.24, and 785.4 but only if at least one of the following vascular disease codes is also present: 250.7, 440.2, 440.21, 440.22, 440.23 |
