# Supplementary material for: Associations between specialty care and improved outcomes among patients with diabetic foot ulcers
Source: PLoS One. 2023 Dec 19;18(12):e0294813. doi: 10.1371/journal.pone.0294813 (PMC10729988; doi:10.1371/journal.pone.0294813)
Supplement: S2 Table — (DOCX) [file pone.0294813.s008.docx]

| **S2 Table. Covariate balance before and after propensity score weighting for each stratum.** | | | | | | | | |  |
| --- | --- | --- | --- | --- | --- | --- | --- | --- | --- |
| **Covariates** | **Means after weighting** | | | | **Means before weighting** | | | |  |
|  | **Never** | **Before entry into stratum** | **After entry into stratum** | **p-value*** | **Never** | **Before entry into stratum** | **After entry into stratum** | **p-value*** |  |
| **Early Stage Stratum** | | | | | | | | |  |
| *Sociodemographics* | | | | | | | | |  |
| Age | 72.95 | -- | 72.84 | 0.2952 | 73.98 | -- | 72.84 | <0.001 |  |
| Female | 0.48 | -- | 0.48 | 0.9981 | 0.45 | -- | 0.48 | <0.001 |  |
| Race & Ethnicity |  |  |  |  |  |  |  |  |  |
| Black | 0.12 | -- | 0.12 | 0.9980 | 0.14 | -- | 0.12 | <0.001 |  |
| Other/unknown | 0.04 | -- | 0.04 | 0.9995 | 0.04 | -- | 0.04 | 0.5743 |  |
| Rurality |  |  |  |  |  |  |  |  |  |
| Rural | 0.16 | -- | 0.16 | 0.9984 | 0.2 | -- | 0.16 | <0.001 |  |
| *Comorbidities* | | | | | | | | |  |
| History of peripheral vascular disease | 0.83 | -- | 0.83 | 0.9999 | 0.85 | -- | 0.83 | <0.001 |  |
| History of myocardial infarction | 0.25 | -- | 0.25 | 0.9996 | 0.3 | -- | 0.25 | <0.001 |  |
| History of uncomplicated diabetes | 0.13 | -- | 0.13 | 0.9977 | 0.19 | -- | 0.13 | <0.001 |  |
| History of renal disease | 0.32 | -- | 0.32 | 0.9986 | 0.46 | -- | 0.32 | <0.001 |  |
| History of eye disease | 0.31 | -- | 0.31 | 0.9995 | 0.27 | -- | 0.31 | <0.001 |  |
| History of heart disease | 0.79 | -- | 0.79 | 0.9975 | 0.82 | -- | 0.79 | <0.001 |  |
| History of stroke | 0.28 | -- | 0.28 | 0.9986 | 0.36 | -- | 0.28 | <0.001 |  |
| **Osteomyelitis Stratum** | | | | | | | | |  |
| *Sociodemographics* | | | | | | | | |  |
| Age | 70.53 | 70.42 | 70.47 | 0.9387 | 70.56 | 70.71 | 70.03 | 0.0692 |  |
| Female | 0.39 | 0.39 | 0.40 | 0.9999 | 0.36 | 0.39 | 0.44 | <0.001 |  |
| Race & Ethnicity |  |  |  |  |  |  |  |  |  |
| Black | 0.13 | 0.13 | 0.13 | 1.0000 | 0.17 | 0.11 | 0.12 | <0.001 |  |
| Other/unknown | 0.05 | 0.05 | 0.05 | 0.9999 | 0.05 | 0.05 | 0.05 | 0.8788 |  |
| Rurality |  |  |  |  |  |  |  |  |  |
| Rural | 0.19 | 0.18 | 0.18 | 0.9999 | 0.20 | 0.18 | 0.16 | 0.0026 |  |
| **S2 Table (con’t)** | | | | | | | | |  |
| *Comorbidities* | | | | | | | | |  |
| History of peripheral vascular disease | 0.90 | 0.90 | 0.89 | 0.9999 | 0.91 | 0.93 | 0.84 | <0.001 |  |
| History of myocardial infarction | 0.30 | 0.29 | 0.29 | 1.0000 | 0.32 | 0.31 | 0.25 | <0.001 |  |
| History of uncomplicated diabetes | 0.08 | 0.07 | 0.07 | 0.9996 | 0.09 | 0.04 | 0.11 | <0.001 |  |
| History of renal disease | 0.39 | 0.38 | 0.39 | 0.9999 | 0.46 | 0.37 | 0.35 | <0.001 |  |
| History of eye disease | 0.37 | 0.38 | 0.37 | 0.9999 | 0.34 | 0.42 | 0.33 | <0.001 |  |
| History of heart disease | 0.83 | 0.83 | 0.82 | 0.9999 | 0.82 | 0.85 | 0.79 | <0.001 |  |
| History of stroke | 0.31 | 0.30 | 0.31 | 0.9999 | 0.34 | 0.30 | 0.29 | 0.0013 |  |
| **Gangrene Stratum** | | | | | | | | |  |
| *Sociodemographics* | | | | | | | | |  |
| Age | 72.63 | 72.64 | 72.59 | 0.9869 | 72.23 | 72.93 | 72.9 | 0.0532 |  |
| Female | 0.40 | 0.40 | 0.41 | 0.9998 | 0.40 | 0.39 | 0.45 | 0.0018 |  |
| Race & Ethnicity |  |  |  |  |  |  |  |  |  |
| Black | 0.21 | 0.20 | 0.21 | 0.9998 | 0.23 | 0.17 | 0.22 | <0.001 |  |
| Other/unknown | 0.06 | 0.06 | 0.06 | 0.9999 | 0.06 | 0.06 | 0.04 | 0.0795 |  |
| Rurality |  |  |  |  |  |  |  |  |  |
| Rural | 0.16 | 0.16 | 0.16 | 1.0000 | 0.17 | 0.16 | 0.13 | 0.0043 |  |
| *Comorbidities* | | | | | | | | |  |
| History of peripheral vascular disease | 0.99 | 1.00 | 0.99 | 0.9995 | 0.99 | 1.00 | 0.99 | 0.0016 |  |
| History of myocardial infarction | 0.44 | 0.44 | 0.44 | 1.0000 | 0.43 | 0.46 | 0.40 | 0.0042 |  |
| History of uncomplicated diabetes | 0.08 | 0.07 | 0.08 | 1.0000 | 0.10 | 0.04 | 0.08 | <0.001 |  |
| History of eye disease | 0.39 | 0.40 | 0.39 | 0.9999 | 0.34 | 0.47 | 0.35 | <0.001 |  |
| History of renal disease | 0.53 | 0.53 | 0.54 | 0.9999 | 0.55 | 0.51 | 0.53 | 0.0297 |  |
| * The p-values are calculated based on the F-test for continuous variables or the chi-square test for binary variables. | | | | | | | | |  |
