# Supplementary material for: Associations between specialty care and improved outcomes among patients with diabetic foot ulcers
Source: PLoS One. 2023 Dec 19;18(12):e0294813. doi: 10.1371/journal.pone.0294813 (PMC10729988; doi:10.1371/journal.pone.0294813)
Supplement: S3 Table — (DOCX) [file pone.0294813.s009.docx]

| **S3 Table. Inverse propensity score weighted and adjusted hazard ratios for the relationship between specialty care and the primary outcome of ulcer progression, major amputation or death, & the secondary outcome of major amputation or death, censoring for ulcer progression.** | | | |
| --- | --- | --- | --- |
| **Ulcer Severity** | **Specialty Care** | | |
|  | **Never** | **Before entry into stratum** | **After entry into stratum** |
| **Early Stage Stratum*** | | | |
| Persons (%) | 14,488 (28.72) | N/A | 35,950 (71.28) |
| Person-years | 11,150 | – | 64,311 |
| aHR for primary outcome (ulcer progression, major amputation, or death) | 1 (ref) | N/A | 0.37 |
| 95% CI for primary outcome | – | – | 0.36-0.38 |
| aHR for secondary outcome (major amputation or death) | 1 (ref) | N/A | 0.32 |
| 95% CI for secondary outcome | – | – | 0.31-0.33 |
| **Osteomyelitis Stratum**^†^ | | | |
| Persons (%) | 2,255 (27.01) | 3,632 (43.50) | 2,463 (29.50) |
| Person-years | 733 | 2,938 | 3,837 |
| aHR for primary outcome (ulcer progression, major amputation, or death) | 1 (ref) | 0.49 | 0.22 |
| 95% CI for primary outcome | – | 0.46-0.53 | 0.21-0.24 |
| aHR for secondary outcome (major amputation or death) | 1 (ref) | 0.49 | 0.22 |
| 95% CI for secondary outcome | – | 0.46-0.53 | 0.20-0.24 |
| **Gangrene stratum**^‡^ | | | |
| Persons (%) | 2,242 (42.21) | 2,036 (38.33) | 1,034 (19.47) |
| Person-years | 428 | 843 | 1,287 |
| aHR for primary outcome (ulcer progression, major amputation, or death) | 1 (ref) | 0.62 | 0.21 |
| 95% CI for primary outcome | – | 0.58-0.67 | 0.19-0.23 |
| aHR for secondary outcome (major amputation or death) | 1 (ref) | 0.62 | 0.21 |
| 95% CI for secondary outcome | – | 0.58-0.67 | 0.19-0.23 |
| * Covariates used to calculate propensity score weights include age, sex, race/ethnicity, rurality, eye disease, heart disease, myocardial infarction, peripheral vascular disease, renal disease, stroke and uncomplicated diabetes.  ^†^ Covariates used to calculate propensity score weights include age, sex, race/ethnicity, rurality, eye disease, heart disease, myocardial infarction, peripheral vascular disease, renal disease, stroke and uncomplicated diabetes.  ^‡^ Covariates used to calculate propensity score weights include age, sex, race/ethnicity, rurality, eye disease, myocardial infarction, peripheral vascular disease, renal disease, and uncomplicated diabetes. | | | |
